# Tumor and germline testing with next generation sequencing in epithelial ovarian cancer: a prospective paired comparison using an 18‐gene panel

**DOI:** 10.1002/1878-0261.70136

**Published:** 2025-10-05

**Authors:** Elisabeth Spenard, Cristina Mitric, Melanie Care, Tracy L. Stockley, Raymond H. Kim, Jeanna McCuaig, Blaise Clarke, Laura Ranich, Clare Sheen, Sarah E. Ferguson, Liat Hogen, Taymaa May, Marcus Q. Bernardini

**Affiliations:** ^1^ Division of Gynecologic Oncology University of Toronto Canada; ^2^ Division of Gynecologic Oncology Princess Margaret Cancer Centre, University Health Network Canada; ^3^ Institute of Health Policy Management and Evaluation, University of Toronto Canada; ^4^ Division of Gynecologic Oncology Laval University Québec City Canada; ^5^ Laboratory Medicine Program, Division of Clinical Laboratory Genetics University Health Network Canada; ^6^ Department of Molecular Genetics University of Toronto Canada; ^7^ Department of Laboratory Medicine and Pathobiology University of Toronto Canada; ^8^ Bhalwani Familial Cancer Clinic Princess Margaret Cancer Centre, University Health Network Canada; ^9^ Division of Medical Oncology Department of Medicine, University of Toronto Canada; ^10^ Division of Gynecologic Oncology Department of Obstetrics and Gynecology, Brigham and Women's Hospital, Dana Farber Cancer Institute, Harvard Medical School Boston USA

**Keywords:** epithelial ovarian cancer, multigene tumor‐testing, next generation sequencing (NGS), somatic testing, paired testing

## Abstract

Genetic testing in epithelial ovarian cancer (EOC) in Ontario includes germline next‐generation sequencing (NGS) for 19 genes. Additionally, tumor tissue undergoes reflex NGS testing for *BRCA1/2* to assess eligibility for PARPi. Although parallel testing confers advantages, this model duplicates healthcare resources. Here, we prospectively assessed the feasibility of tumor‐first multigene testing by comparing tumor tissue with germline testing of peripheral blood. An 18‐gene NGS panel was used to test tumor and germline DNA (*n* = 106 patients). In 26 patients, 27 tumor Tier I or II variants were identified, with 16/27 (59%) being germline pathogenic variants (PV) (13 *BRCA1/2*; 3 other genes) and 11/27 (41%) somatic variants (9 *BRCA1/2*; 2 other). In 51/106 patients, there were no tumor variants (excluding *TP53*), of which one patient had a germline *BRCA1* copy number variant deletion in exon 12. Tumor‐first testing detected variant‐positive and variant‐negative germline cases in 105/106 patients (99.1%). Among 50 *BRCA*‐negative patients, 14/50 (28%) were homologous recombination deficiency (HRD)‐positive. Therefore, we demonstrate that multigene NGS tumor‐testing is effective in identifying germline variants in EOC with a < 1% false‐negative rate.

## Graphical abstract

Genetic testing in epithelial ovarian cancer includes both germline and tumor‐testing. This approach often duplicates resources. The current prospective study assessed the feasibility of tumor‐first multigene testing by comparing tumor tissue with germline testing of peripheral blood using an 18‐gene NGS panel in 106 patients. This method for identifying germline variants is feasible and effective with a < 1% false‐negative rate.

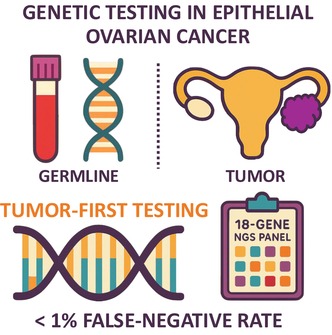

**Abbreviations**
CA125: cancer antigen 125
CNV: copy number variant
ECOG: Easters Cooperative Oncology Group performance status
eFHQ: electronic family history questionnaire
EOC: epithelial ovarian cancer
FFPE: formalin‐fixed paraffin‐embedded
FIGO: International Federation of Obstetrics and Gynecology
GATK: Genome Analysis Toolkit
GIS: genomic instability score
HRD: homologous recombination deficiency
IR: interventional radiology
LGSC: low‐grade serous carcinoma
LP: likely pathogenic variant
MLPA: multiple ligation probe amplification
NACT: neoadjuvant chemotherapy
NGS: next generation sequencing
PARPi: poly ADP‐ribose polymerase inhibitor
PV: pathogenic variant
UHN: University Health Network
VAF: variant allele fraction
VUS: variant of uncertain significance

## 1 Introduction

Epithelial ovarian cancer (EOC) affects 1.5% of women and is the fifth leading cause of cancer death among females in North America [1, 2]. Genetic pathogenic variants (PV) are responsible for 20–30% of EOC, most commonly occurring in the *BRCA1*/2 genes [3, 4]. Other causative genes include those involved in mismatch repair (*MLH1, MSH2, MSH6, PMS2*, and *EPCAM*) or the Fanconi‐anemia pathway (*RAD51C, RAD51D, BRIP1*, and *PALB2*). Although PV in other genes such as *ATM, CDH1, CHEK2, PTEN*, and *TP53* have been observed in families with ovarian cancer, this data remain controversial [5, 6].

Information about both somatic and germline status is relevant for patients and their families, as knowledge of *BRCA1/2* status can impact treatment options. For instance, the use of poly ADP‐ribose polymerase inhibitors (PARPi) has been shown to significantly increase progression‐free and overall survival [7, 8, 9]. Similarly, knowledge about germline PV in EOC risk genes can provide cancer risk‐reduction and surveillance options for patients and their relatives [10, 11, 12]. Risk‐reducing bilateral salpingo‐oophorectomy can lead to a 90% decreased risk of EOC [11, 13]. Ovarian cancer patients with a germline *BRCA1/2* mutation have a 50% chance of a first‐degree relative also having the mutation.

The current standard of care for genetic testing in EOC in Ontario includes germline next‐generation sequencing (NGS) genetic testing for 19 genes including *ATM, BARD1, BRCA1, BRCA2, BRIP1, CDH1, CHEK2, EPCAM, MLH1, MSH2, MSH6, PALB2, PMS2, PTEN, RAD51C, RAD51D, HOXB13, STK11*, and *TP53* [14]. In addition, tumor tissue from all EOC patients undergoes reflex testing for *BRCA1/2* to assess eligibility for PARPi [14]. For *BRCA1/2*, it is known that approximately 5–7% of EOC have acquired somatic PVs present only in tumor tissue, which cannot be detected through germline testing [7, 8]. To ensure optimal genetic testing and care for all EOC patients, the parallel approach of multigene germline testing and somatic *BRCA* gene testing is increasingly recommended by gynecologic oncology societies [8].

Although parallel germline and tumor‐testing confers advantages, the current model in Ontario duplicates healthcare resources by requiring NGS genetic testing of both germline and tumor tissue samples in EOC. At the same time, evidence from our center showed that a tumor‐first approach has a high sensitivity to detect both somatic and germline variants in *BRCA1/2* [15]. The objective of this study was to expand on our *BRCA1/2* evidence to a multigene panel and prospectively explore the feasibility of multigene tumor‐testing in EOC by comparing tumor tissue test results with results from germline testing of peripheral blood. We hypothesized that NGS multigene panel tumor‐testing can accurately identify germline variants, and a multigene panel tumor‐first triage model for EOC can be considered.

## 2 Methods

A single‐center prospective single‐arm cohort study was conducted between October 2021 and October 2023 at the Princess Margaret Cancer Center/University Health Network (UHN) in Toronto, Ontario, Canada. This study conformed to the standards of the Declaration of Helsinki. UHN Research Ethics Board approval was obtained (#20‐5852).

### 2.1 Participants

Patients with suspected advanced epithelial ovarian cancer (EOC) were approached to participate at their initial gynecologic oncology visit prior to initial surgery or chemotherapy treatment, and consent was obtained from all eligible patients by a research team member. The study inclusion criterion was histology‐confirmed EOC, incorporating serous, clear cell, endometrioid, mucinous, and mixed adenocarcinoma histology. Exclusion criteria were lack of informed consent, age under 18, lack of EOC or cancer confirmation, low‐grade serous histology, and patients receiving treatment at a different institution. Patients were also excluded if the tissue or DNA sample was insufficient or unavailable to perform testing. Patient demographics and clinical data (including age, tumor marker value, initial treatment, family history, pathological stage, and histological subtype), as well as testing results, were collected using the REDCap platform. All participants provided written informed consent prior to inclusion in the study.

### 2.2 Samples and DNA extraction

For tumor‐testing, a formalin‐fixed paraffin‐embedded (FFPE) sample was reviewed by a pathologist with expertise in gynecologic oncology at the time of initial surgery or biopsy to confirm an EOC diagnosis and ensure sufficient tumor cellularity for NGS (> 20%). Tumor enrichment prior to DNA extraction was performed by sampling 2 × 1 mm cores in a tumor‐rich region, or by microdissection of sections (8 slides at 7‐um thickness). FFPE samples were digested overnight in Proteinase K (20 mg·mL^−1^) and DNA extracted using a magnetic bead purification method (Maxwell FFPE Plus DNA Purification Kit; Promega, Madison, WI, USA) on an automatic extractor (Maxwell 16; Promega). As per institutional policy, if initial testing failed due to insufficient tumor cellularity or insufficient extracted DNA, a second attempt was undertaken on a surgical specimen or repeat biopsy.

For germline tissue, genomic DNA was extracted from peripheral blood samples using QIAsymphony DNA Midi kit (Qiagen, Germantown, MD, USA). DNA concentration for both tumor and germline DNA was evaluated using fluorometry (Qubit dsDNA Assay Kit on the Qubit 2.0 Fluorometer; Thermo Fisher Scientific, Waltham, MA, USA).

### 2.3 NGS testing

NGS testing was performed at our institutional clinical molecular pathology laboratory (UHN Genome Diagnostics) with experience in NGS multigene testing including *BRCA1/2* and other EOC genes. DNA from tumor and germline tissues was analyzed on a custom NGS panel including 18 EOC genes (*BRCA1, BRCA2, MLH1, MSH2, MSH6, PMS2, EPCAM, RAD51C, RAD51D, BRIP1, ATM, BARD1, CDH1, CHEK2, PALB2, PTEN, STK11*, and *TP53*). NGS panel testing was clinically validated on DNA extracted from peripheral blood leukocytes (germline) and from tumor tissue (both FFPE and cytology specimens) [15]. Note that genes tested on tumor excluded the *HOXB13* gene given the typical association of *HOXB13* with prostate and lack of association with ovarian cancer [14, 15]. Testing was performed as previously described [15]. Briefly, hybridization capture libraries (SureSelect XT, Agilent Technologies, Santa Clara, CA, USA) were sequenced on the Illumina platform (NextSeq 500; Illumina, San Diego, CA, USA). To ensure appropriate variant detection, all samples had a minimum read depth of 100× (tumor) and 25× (germline) on target regions. Data analysis used custom bioinformatic analysis with Burrows‐Wheeler Aligner [16] and Genome Analysis Toolkit (GATK) tools. Variant calling used Varscan [17], and copy number variants (CNV) were assessed by CNVkit [18] and DECoN [19]. For any CNVs identified by NGS testing as a possible deletion (CNVkit log_2_ ratio less than −0.8, DECoN reads ratio less than 0.8) or duplication (CNVkit log_2_ ratio greater than 0.8, DECoN reads ratio greater than 1.2), verification was performed using the multiple ligation probe amplification (MLPA) kits P002, P087, and P090 (MRC‐Holland, Amsterdam, The Netherlands). The assay was previously demonstrated to detect single exon level deletions or duplications in *BRCA1* and *BRCA2* [15].

### 2.4 Variant classifications

Identified variants were filtered to remove technical artifacts, and benign or likely benign variants (Alissa Clinical Informatic Platform, Agilent). Tumor variants were classified using the Joint Consensus Recommendation of the Association for Molecular Pathology, American Society of Clinical Oncology, and College of American Pathologists [20] and assigned to one of the following classifications: Tier I (variants with strong clinical significance), Tier II (variants with potential clinical significance), or Tier III (variants with unknown clinical significance). Tier IV benign or likely benign variants were removed by filtering as described.

Germline variants were classified using the 2015 guidelines from the American College of Medical Genetics and Genomics and the Association for Molecular Pathology [21]. Variants classified as pathogenic (P), likely pathogenic (LP), or of uncertain significance (VUS) were reported. Benign or likely benign variants were not reported.

### 2.5 Homologous recombination

Homologous recombination deficiency (HRD) analysis was performed on *BRCA‐negative* tumor tissue at the Myriad Genetic Laboratories using the Myriad myChoice HRD Plus assay [22] and standard protocol to obtain a genomic instability score (GIS). A GIS threshold of greater than or equal to 42 was used to define a positive HRD status [2].

### 2.6 Sample handling

A research coordinator ensured compliance with the screening pathway and dual somatic and germline testing being performed. Fig. S1 provides an overview of the patient care trajectory from study enrolment to follow‐up post treatment, and Table S1 provides an overview description of the testing strategies.

### 2.7 Statistical analysis

Descriptive statistics were used to summarize participant characteristics. Categorical variables were summarized using counts and percentages, whereas continuous variables were summarized using medians and ranges.

## 3 Results

**Fig. 1:**
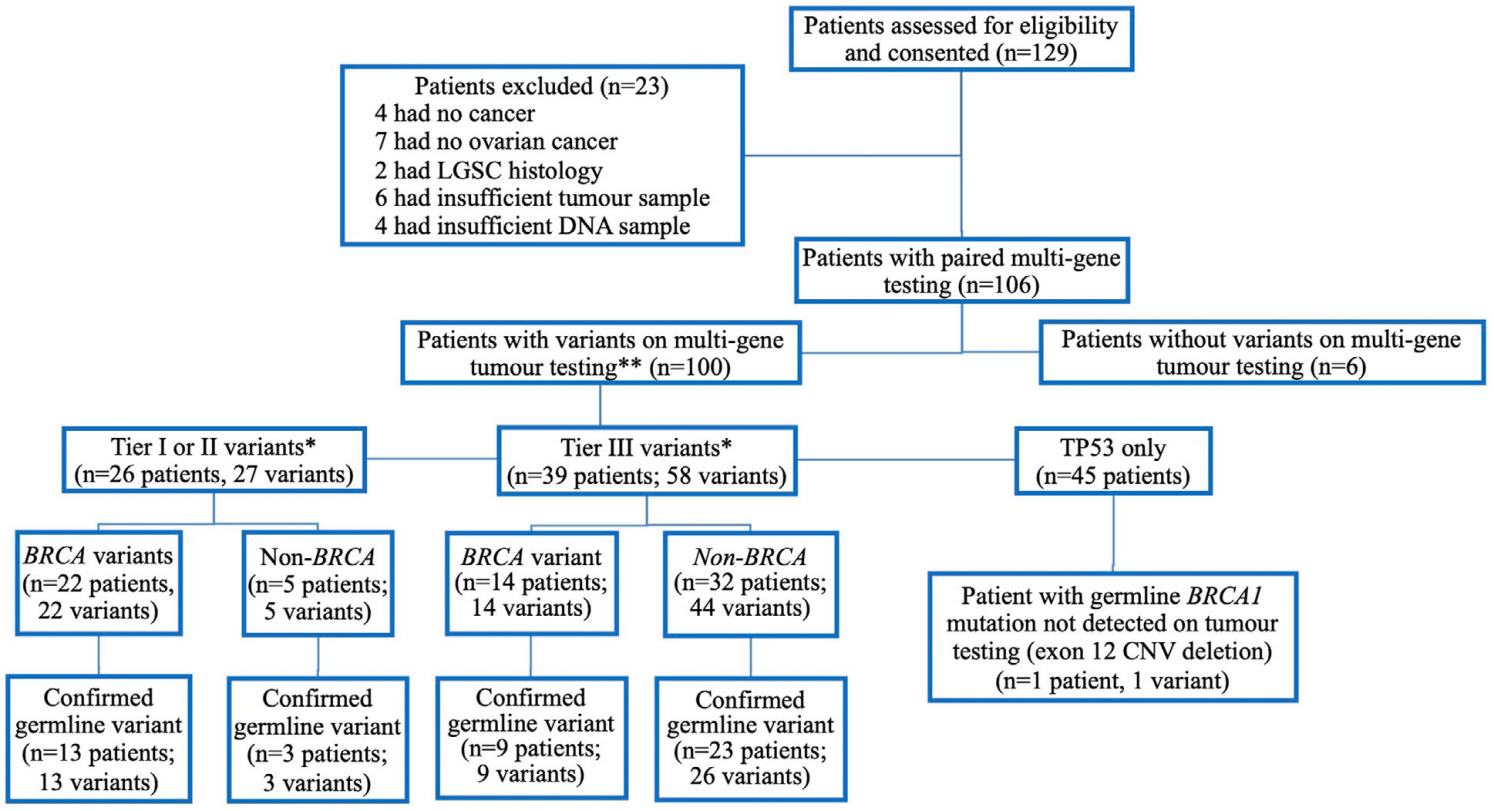
Enrollment and outcome diagram. LGSC, low‐grade serous carcinoma; LP, likely pathogenic variant; *n*, number; PV, pathogenic variant; VUS, variant of unknown significance. This Figure excluded TP53 tumor variants. Variants were classified according to scheme of Richards et al. 2015 [21].

A total of 129 patients with presumed advanced ovarian cancer were consented for the study, and 23 were excluded (Fig. 1), leaving 106 patients with EOC that completed paired tumor and germline 18‐gene NGS testing and were included in the final analysis. Their demographic characteristics are reported in Table 1. Among the 106 tumors tested, six (5.7%) had no variants identified. In the remaining 100 tumors (94.3%), there were a total of 185 variants identified: 100 *TP53* variants (not classified), 27 Tier I/II variants, and 58 Tier III variants. Twenty‐two large deletions or duplications were identified on tumor, ranging in size from a single exon to whole gene; six of them were identified in *BRCA1/2*.

**Table 1:** Patient demographics and clinical information. CA125, cancer antigen 125; cm, centimeter; ECOG, Eastern Cooperative Oncology Group performance status; FIGO, International Federation of Obstetrics and Gynecology; *n*, number.

Patient characteristics	*n* = 106
Age in years: median (range)	63 (46–90)
Stage FIGO 2018: *n* (%)	
I–IIB	5 (4.7%)
IIIB	16 (15.1%)
IIIC	49 (46.2%)
IVA	14 (13.2%)
IVB	22 (20.8%)
CA125 level: median (range)	967 (20, 18 700)
ECOG status: *n* (%)	
0	70 (66.7%)
1	28 (26.7%)
2	5 (4.7%)
3	2 (1.9%)
First‐line treatment: *n* (%)	
Primary debulking surgery	49 (46.2%)
Neoadjuvant chemotherapy, interval surgery	33 (31.1%)
Neoadjuvant chemotherapy, no surgery	22 (20.8)
Supportive care	2 (1.9%)
Surgical outcome: *n* (%)	
Optimal (< 1 cm)	72 (67.9%)
Suboptimal (> 1 cm)	6 (5.7%)
Open and close	4 (3.8%)
No surgery	24 (22.6%)
Histology: *n* (%)	
High‐grade serous	97 (91.5%)
Clear cell	6 (5.7%)
Endometrioid	2 (1.9%)
Other	1 (0.9%)
Pathology sample: *n* (%)	
Biopsy	74 (69.8%)
Primary debulking surgery	28 (26.4%)
Interval debulking surgery	4 (3.8%)
Family history of cancer: *n* (%)	88 (83.0%)
Prior cancer history: *n* (%)	17 (16.0%)

For the 27 Tier I/II tumor variants, germline DNA testing confirmed 16/27 (59.3%) to be of germline origin (P or LP variants), while the remaining 11/27 (40.7%) were somatic variants. For the 58 Tier III tumor variants, germline DNA testing confirmed 35/58 (60.3%) to be of germline origin (all VUS), whereas the remaining 23/58 (39.7%) were somatic variants. Among the 106 total patients, 51/106 (48.1%) had no variant identified on tumor‐testing other than *TP53*, and 50/51 (98.0%) of these had confirmatory negative results on paired germline testing. One patient with *TP53* as the sole somatic variant was found to have an exon 12 *BRCA1* pathogenic deletion on subsequent germline testing. This patient's family history was significant for three second‐degree relatives diagnosed with cancer (liver, skin, and prostate). Fig. 1 illustrates the outcome and correlation for tumor‐testing followed by germline testing for all patients, highlighting the presence of *BRCA 1/2* variants (*n* = 106). Tumor‐first testing correctly detected variant‐positive and variant‐negative germline cases in 105/106 (99.1%).

**Fig. 2:**
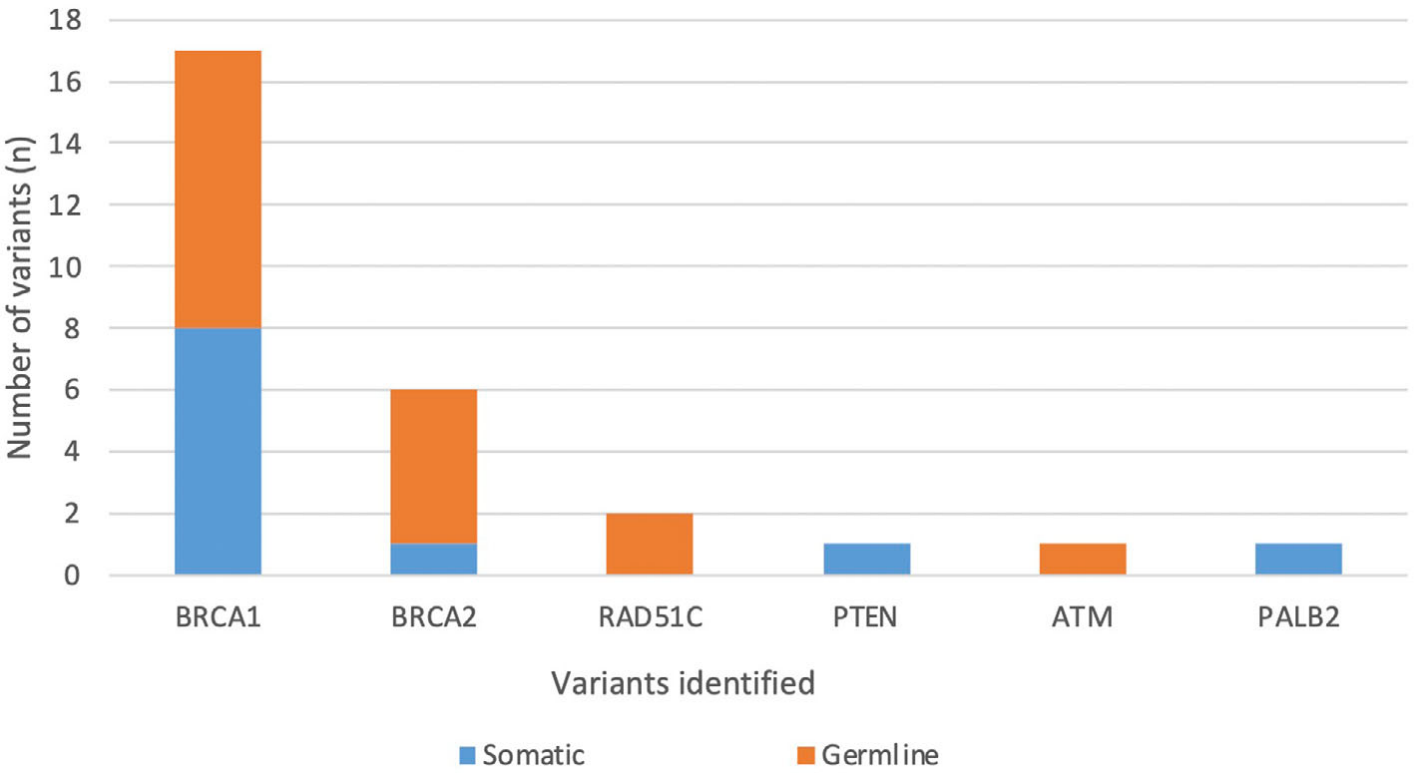
Variants identified on paired tumor‐germline testing (excluding *TP53* variants) (*n* = 106). *n*, number. This Figure included likely pathogenic and pathogenic germline variants, as well as Tiers I and II somatic variants.

**Fig. 3:**
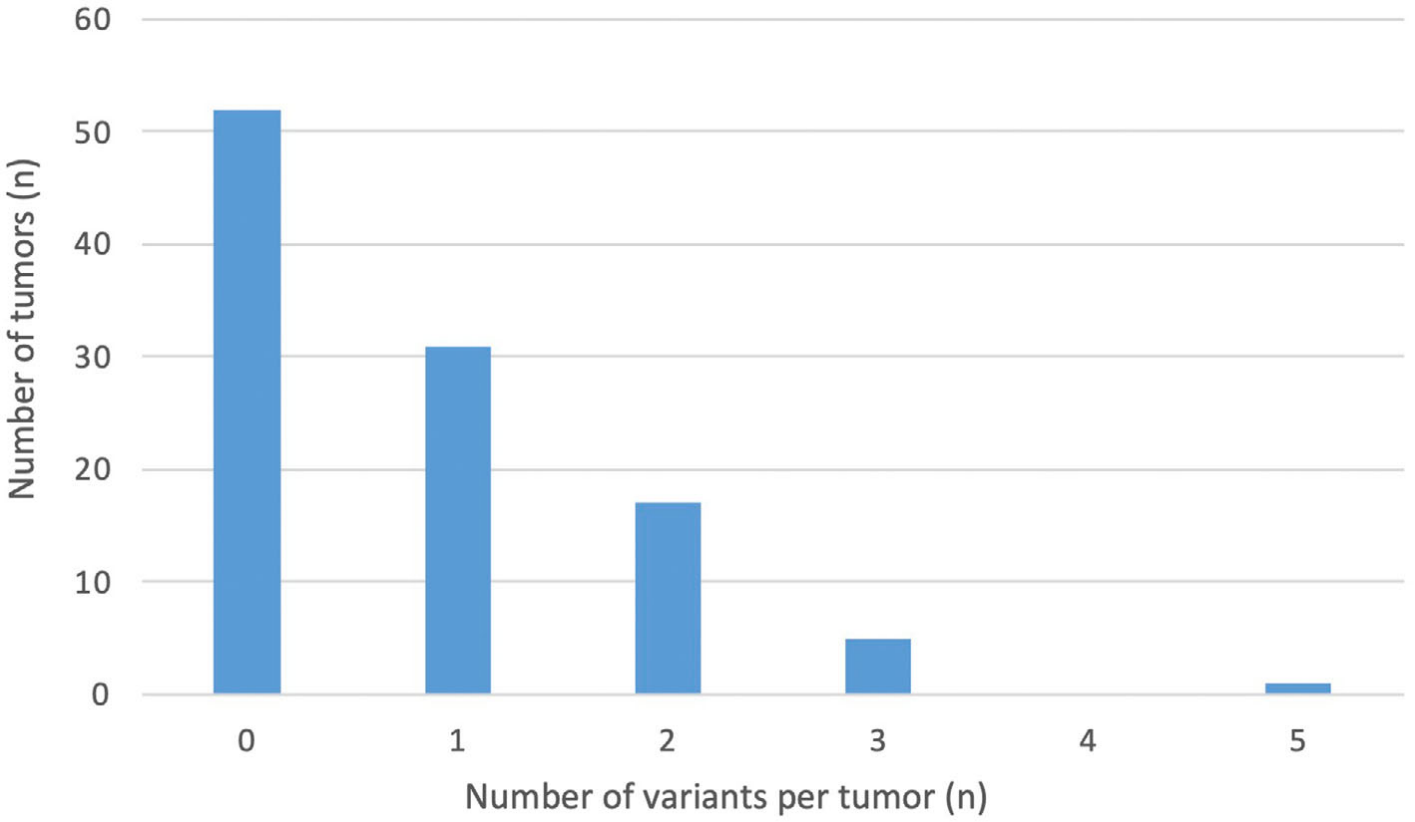
Tumor variant burden (excluding *TP53* variants) (*n* = 106). *n*, number. This Figure excluded TP53 tumor variants.

**Fig. 4:**
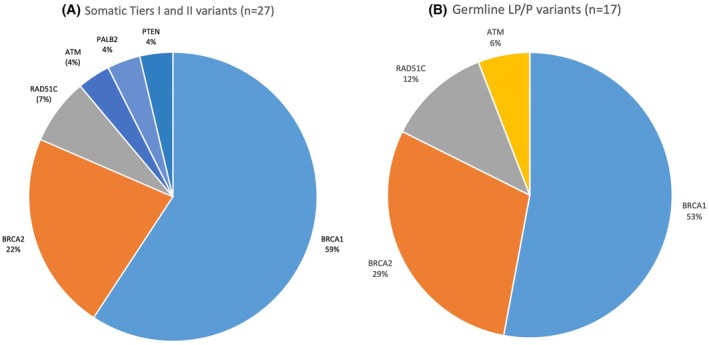
Variant description (A) Somatic Tiers I and II variants (*n* = 27). (B) Germline likely pathogenic and pathogenic (LP/P) variants (*n* = 17). LP, likely pathogenic variant; *n*, number; PV, pathogenic variant. This Figure included likely pathogenic and pathogenic germline variants, as well as Tiers I and II somatic variants. Variants were classified according to scheme of Richards et al. 2015 [21].

A total of 28 clinically significant variants were identified using germline and tumor‐testing: *BRCA1* (17), *BRCA2* (6), *RAD51C* (2), *ATM* (1), *PALB2* (1), and *PTEN* (1). Fig. 2 illustrates the proportion of P/LP germline variants compared to Tier I/II somatic variants. Most patients were found to have one or two tumor variants after excluding *TP53* (Fig. 3). P/LP (germline, *n* = 17) or Tier I/II (tumor, *n* = 27) variants were most frequently observed in *BRCA1*, with an incidence of 53% and 59%, respectively (Fig. 4A,B). Other common P/LP germline variants include *BRCA2* (29%), *RAD51C* (12%), and *ATM* (6%). Other common Tier I/II somatic variants include *BRCA2* (22%) and *RAD51C* (7%). For somatic Tier III variants, *BRCA2* alterations were the most common (19%), followed by *MSH6* (17%), and then *STK11* (12%) (Fig. S2A). In the germline, VUS (*n* = 35) were most identified in *BRCA2* and *MSH6* (23% each), followed by *PMS2*, *PALB2*, and *ATM* (all 9%) (Fig. S2B).

HRD testing was performed among 50 of 83 *BRCA*‐negative patients (60.2%). Reasons for not undergoing HRD testing are reported in Table S2. There were 14/50 (28%) patients with positive HRD testing (GIS ≥ 42), 30/50 (60%) with negative results, and 6/50 (12%) with inconclusive results. Among the 14 patients with positive HRD scores (Table 2), two (14.3%) had pathogenic variants: one with a *RAD51C* germline PV identified as a Tier II tumor variant, and one with a *PALB2* Tier II somatic variant. Among the 30 patients with negative HRD scores (Table 3), one (3.3%) had a *RAD51C* LP variant identified as a Tier II tumor variant; this patient had a GIS of 40.

**Table 2:** Positive Myriad HRD status compared to NGS multigene paired testing (*n* = 14/50). GIS, Genomic Instability Score (HRD positive score ≥ 42); HRD, Homologous recombination deficiency; n/a, not applicable; NGS, Next generation sequencing; PV, pathogenic variant; VAF, Variant allele fraction; VUS, Variant of uncertain significance.

Patient #	HRD status	GIS score	NGS germline testing	NGS tumor‐testing (tier)^a^	Variant (cDNA)	Variant (protein)	Tumor VAF (%)
3	Positive	48	Negative	*TP53*	c.388C>G	p. (Leu130Val)	75.6
6	Positive	63	*PMS2* (VUS)	*PMS2* (III)	c.53 T>C	p.(Ile18Thr)	46.3
				*TP53*	c.517 T>C	p.(Val173Leu)	61.4
16	Positive	57	*STK11* (VUS)	*STK11* (III)	c.976C>A	p. (Pro326Thr)	51.4
				*TP53*	c.838delA	p.(Arg280Glufs*65)	33.1
23	Positive	51	*RAD51D* (VUS)	*RAD51D* (III)	c.823C>T	p.(Arg275Trp)	86.3
				*MSH2* (III)	c.2005 + 7A>T	p.(?)	5.6
				*TP53*	c.743G>A	p.(Arg248Gln)	76.2
26	Positive	72	*CHEK2* (VUS)	*CHEK2* (III)	c.460A>C	p.(Asn154His)	8.4
				*BRCA2* (III)	NA	Full gene deletion	n/a
				*ATM* (III)	NA	Full gene duplication	n/a
				*TP53*	1025G>C	p.(Arg342Pro)	77.5
34	Positive	58	Negative	*TP53*	c. 747G>T	p.(Arg249Ser)	70.9
39	Positive	43	Negative	*PALB2* (II)	NA	exon 1 deletion	n/a
				*CHEK2* (III)	NA	exon 2–15 duplication	n/a
				*TP53*	c.646G>A	p.(Val216Met)	74.8
50	Positive	75	*BRCA2* (VUS)	*BRCA2* (III)	c.7435 + 1‐G>A	p.(?)	47.9
			*MSH6* (VUS)	*MSH6* (III)	c.3760G>C	p.(Glu1254Gln)	35.9
				*TP53*	c.714dupT	p.(Asn239^a^)	66.8
57	Positive	59	Negative	*TP53*	c.395A>G	p.(Lys132Arg)	89.8
64	Positive	60	*MSH2* (VUS)	*MSH2* (III)	c.2516A>G	p.(His839Arg)	64.4
			*MSH6* (VUS)	*MSH6* (III)	c.107C>T	p.(Ala36Val)	66.5
				*TP53*	c.375G>A	p.(Thr125Thr)	57.2
74	Positive	50	*MSH2* (VUS)	*MSH2* (III)	c.421A>G	p.(Met141Val)	58.4
				*TP53*	c.993 + 1G>A	p.(?)	23.1
79	Positive	55	Negative	*TP53*	c.830G>T	p.(Cys277Phe)	70.5
82	Positive	72	*RAD51C* (VUS)	*RAD51C* (III)	c.571 + 4A>G	p.(?)	56
				*TP53*	c.949C>T	p.(Gln317^a^)	17.5
99	Positive	54	*RAD51C* (PV)	*RAD51C* (II)	c.706‐2A>G	p.(?)	77.9
				*TP53*	c.920‐2A>G	p.(?)	45.2

^a^ TP53 variants not classified.

**Table 3:** Negative Myriad HRD status compared to NGS multigene testing (*n* = 30/50). GIS, Genomic Instability Score (HRD positive score ≥ 42); HRD, Homologous recombination deficiency; n/a, not applicable; NGS, Next generation sequencing; PV, pathogenic variant; VAF, Variant allele fraction; VUS, Variant of uncertain significance.

Patient #	HRD status	GIS score	NGS germline testing	NGS tumor (tier)^a^	Variant (cDNA)	Variant (protein)	Tumor VAF (%)
4	Negative	1	Negative	*TP53*	c.388C>G	p.(Arg273His)	36.8
10	Negative	20	Negative	*BRCA1* (III)	c.4193A>T	p.(Asp1398Val)	39.2
				*STK11* (III)	NA	Whole gene deletion	n/a
13	Negative	12	*BRCA2* (VUS)	*BRCA2* (III)	c.1127 T>G	p.(Phe376Cys)	66
				*TP53*	c.488A>G	p.(Tyr163Cys)	94
14	Negative	21	Negative	*TP53*	c.640delC	p.(His214Ilefs*33)	36.1
19	Negative	40	*RAD51C* (LP)	*RAD51C* (II)	c.701C>G	p.(Ser234^a^)	83.9
				*TP53*	c.818G>A	p.(Arg273His)	50.9
22	Negative	9	Negative	*TP53*	c.721dupT	p.(Ser241Phefs*23)	62.8
37	Negative	23	*CDH1* (VUS)	*CDH1* (III)	c.2296‐4A>G	c.2296‐4A>G	9.7
				*PALB2* (III)	NA	Whole gene duplication	n/a
				*TP53*	c.713G>T	p.(Cys238Phe)	80.3
42	Negative	30	*BRCA2* (VUS)	*BRCA2* (III)	c.9501 + 3A>T	p.(?)	59.4
				*MSH2* (III)	NA	Whole gene duplication	n/a
				*MSH6* (III)	NA	Whole gene duplication	n/a
				*STK11* (III)	NA	Whole gene deletion	n/a
				*EPCAM* (III)	NA	Whole gene duplication	n/a
				*TP53*	c.734G>T	p.(Gly245Val)	84.1
43	Negative	34	Negative	*TP53*	c.524G>A	p.(Arg175His)	61.6
51	Negative	22	Negative	Negative	—	—	—
52	Negative	22	Negative	*TP53*	c.524G>A	p.(Arg175His)	94.7
61	Negative	41	Negative	*TP53*	c.851_852delCA	p.(Thr284Argfs*21)	53.3
63	Negative	10	Negative	*TP53*	c.625A>T	p.(Arg209^a^)	35.2
65	Negative	21	*BRCA2* (VUS)	*BRCA2* (III)	c.5278 T>G	p.(Ser1760Ala)	45.2
68	Negative	17	Negative	*TP53*	c.818G>A	p.(Arg273His)	10.4
69	Negative	23	Negative	*TP53*	c.841G>A	p.(Asp281Asn)	78.7
80	Negative	21	Negative	*TP53*	c.743G>A	p.(Arg248Gln)	47.1
84	Negative	35	Negative	*TP53*	c.818G>T	p.(Arg273Leu)	38.3
87	Negative	22	Negative	Negative	—	—	—
88	Negative	29	Negative	*TP53*	c.659A>G	p.(Tyr220Cys)	73.3
90	Negative	12	Negative	*TP53*	c.916C>T	p.(Arg306^a^)	58.5
92	Negative	15	Negative	*TP53*	c.145delG	p.(Asp49Ilefs*74)	56.5
95	Negative	15	*PMS2* (VUS)	*PMS2* (III)	c.1567 T>A	p.(Ser523Thr)	26.2
				*TP53*	c.723delC	p.(Cys242Alafs*5)	31.1
96	Negative	39	*BRCA2* (VUS)	*BRCA2* (III)	c.6541G>C	p.(Gly2181Arg)	56.4
				*TP53*	c.838A>G	p.(Arg280Gly)	27.4
97	Negative	12	*BRCA2* (VUS)	*BRCA2* (III)	c.1903G>C	p.(Asp536His)	6.8
				*TP53*	c.503A>G	p.(His168Arg)	90.8
98	Negative	16	Negative	*TP53*	c.517G>T	p.(Val173Leu)	13.1
101	Negative	24	Negative	*TP53*	c.637C>T	p.(Arg213^a^)	52.4
102	Negative	36	Negative	*TP53*	c.268delT	p.(Ser90Pro*33)	24
103	Negative	22	Negative	*TP53*	c.574C>T	p.(Gln192^a^)	54.2
105	Negative	10	Negative	*TP53*	c.736A>G	p.(Met246Val)	76.2

^a^ TP53 variants not classified.

## 4 Discussion

The current prospective study performed paired germline‐tumor 18‐gene panel NGS testing for 106 patients with EOC. The results showed that tumor panel testing is both feasible and reliable in detecting germline variants, as tumor‐testing correctly identified variant‐positive and variant‐negative germline cases in 99.1% of cases. In terms of *BRCA1/2* variants, the current study supports our previously reported retrospective data of matched tumor and germline samples from 200 EOC patients, where we demonstrated a 100% concordance rate between tumor and germline *BRCA1/2* NGS testing [15]. For other EOC‐risk genes, specifically *RAD51, ATM, PTEN*, and *PALB2*, tumor‐testing correctly identified all germline variants in the current study. This prospective data support our previous retrospective study, which also identified 10 additional variants (*BRIP1, MSH2, RAD51C*, and *RAD51D*) in the tumor samples with subsequent germline confirmation [15]. Several studies in the literature looked at tumor and germline paired testing using a panel extending to other genes associated with EOC and showed promising results about the feasibility of multigene tumor‐testing in EOC [23, 24, 25, 26, 27, 28]. In other words, the current study provides important prospective ground for reinforcing evidence about the reliability of integrated tumor‐germline testing in EOC. A multigene tumor‐testing triage is important to consider, as it is recognized that a tumor‐first approach can help minimize patients lost to follow‐up for germline‐only testing [10].

The current study provides evidence that a tumor‐first approach for genetic panel testing in EOC can reduce duplicate testing. Broad gene panel tumor‐testing identified that 48.1% of patients had no variants (excluding *TP53*), and concurrent germline testing confirmed that no additional germline variants were present. This suggests that patients with tumor *TP53* variants in isolation may not require genetic counseling and follow‐up germline testing, as the probability of identifying a relevant germline variant in any gene included on the tumor‐testing panel is low. The exception to this is the presence of a family history of ovarian cancer or Li‐Fraumeni syndrome, or patients younger than 30 years [29]. If a relevant tumor variant is identified, subsequent germline testing would be performed on this subgroup to provide information about variant origin (germline versus tumor) and need for genetic testing for at‐risk relatives in the event of germline confirmation. If no variant is identified, then this subgroup (i.e., approximately half of the EOC patients) would not require follow‐up germline analysis, substantially reducing duplicate testing. This model is expected to decrease workloads for both molecular genetics laboratories and hereditary cancer clinics. This is important given that wait times for genetics assessment are a recognized barrier to accessing genetic testing in Canada and across North America [30, 31]. Reflex tumor‐testing for *BRCA1/2* in the ovarian cancer population has already been shown to increase access to genetic testing and decrease wait times [32]. Expanding a tumor‐testing program to include all potentially relevant hereditary genes would be expected to demonstrate a similar effect. In addition, expanded tumor‐testing could address raised concerns about high healthcare and societal costs associated with high‐volume duplicate genetic testing in EOC [33, 34, 35].

The current study had a < 1% false‐negative rate in terms of tumor panel ability to correctly categorize germline variant status. The one false negative involved a germline pathogenic single exon 12 deletion in *BRCA1* that was not reported in testing of DNA from the FFPE‐matched tumor sample. Of note, this is a small deletion involving a single exon, which is recognized to be particularly challenging to detect using NGS performed on FFPE samples. The potential for false‐negative results in either tumor or germline testing is an important consideration. This has been of particular concern with respect to single exon copy number variation in tumor‐testing [36]. The results of this and our previous study [15] demonstrate that tumor NGS testing that is optimized for FFPE analysis has the capability to detect most germline variants including single and multi‐exon CNVs. In considering a tumor‐testing program, there may be additional strategies to further increase the detection of these challenging CNVs, for example ensuring optimal DNA quality from FFPE extraction used in NGS testing, performing a review of CNV data close to thresholds to detect potential CNVs, or using multiple CNV callers on NGS data. Concurrent tumor and germline testing for all patients is currently employed by many to maximize detection with the intent to ensure no clinically relevant variants remain undetected. However, there is increasing evidence that appropriately validated tumor‐testing performed in an experienced laboratory is highly sensitive for detecting germline variants [15, 37]. When considering future testing program development, the resources required to complete duplicate testing for every patient need to be carefully weighed against the low probability of false‐negative results.

PARPi treatment is key for improving survival outcomes in EOC [9, 38]. For patients with no detected *BRCA* germline or somatic variant, HRD testing is an important predictor of the PARPi treatment effect [38]. In this study, 50 *BRCA*‐negative patients were tested by HRD analysis (Myriad myChoice CDx HRD), and 14/50 (28%) patients were found to have a positive genomic instability score (GIS). Two of 14 patients were found to have pathogenic variants with our regional approved NGS EOC panel (1 germline PV in *RAD51C*; 1 somatic Tier II variant in *PALB2*), indicating that HRD analysis can possibly identify more patients suitable for PARPi than NGS gene‐level panels alone. However, the identification of one patient with a germline PV in *RAD51C* and a negative GIS (GIS = 40) indicates that multiple strategies remain important to maximize the benefit for patients. According to the PRIMA trial, patients with negative GISs derive less benefit from PARPi maintenance [38]. However, there is emerging evidence regarding the role of other genes involved in the homologous recombinant pathway, for instance *ATM* and *RAD51* status in predicting PARPi efficacy and chemotherapy response [39, 40, 41]. In other words, a broader genetic panel for tumor‐testing can provide useful first‐step information for assessing hereditary risk; however, the immediate utility of identifying non‐BRCA tumor PVs is less clear.

The current study has several advantages. First, it describes evidence for paired comparisons between tumor and germline NGS panel genetic testing in EOC using a prospective approach, this allowing for real‐time comparisons and eliminating recall or selection bias. In addition, it provides a technical platform for tumor multigene NGS testing in EOC using standard methodology based on recommendations from the Association for Molecular Pathology, American Society of Clinical Oncology, and College of American Pathologists for the tumor variants, and the American College of Medical Genetics and Genomics and the Association for Molecular Pathology for the germline variant classification [20, 21].

The study does have several limitations. For instance, the sample size limits conclusions about rare histology EOC subtypes or non‐*BRCA* variants associated with EOC, which are less frequent. This could limit understanding and conclusions about rare pathogenic variants or low‐frequency genes. Similarly, further research focused on specific histological subtypes could further explore the understanding of paired tumor‐germline testing in these rarer subtypes. As well, HRD testing is performed on a subgroup of the sample, and *BRCA* somatic Tiers I and II did not undergo HRD testing. This limits the ability to generate hypotheses correlating HRD testing results and non‐*BRCA* tumor variants. Also, the study focused on the genes part of the Ontario current panel testing; no conclusions can be made regarding emerging or moderate‐penetrance genes not included. Finally, this study was not powered to assess the influence of PV on treatment strategy and survival. A recent study by Kim et al. showed that patients with stage III/IV BRCA mutated high‐grade serous ovarian cancers had improved survival with primary cytoreductive surgery compared with neoadjuvant chemotherapy, which would support the necessity of early genetic screening to streamline the primary management of advanced ovarian cancer [42].

## 5 Conclusion

In conclusion, the results of the current study provide further evidence for consideration of a tumor‐first approach in EOC as the sensitivity of molecular tumor test methods continues to improve. Tumor multigene NGS testing is a reliable method in EOC, as it is feasible, efficient in detecting *BRCA* and non‐*BRCA* variants, can be performed reflexively on biopsy or surgical specimens, and can decrease duplication of genetic testing. Our study illustrates that near half of the patients could potentially avoid germline testing and genetic counseling following negative tumor results, while also carefully considering other guidelines for testing, such as clinical indications (family history) or limitations of tumor‐testing (allele dropout, CNVs, tumor heterogeneity). For those with positive tumor results, subsequent germline testing is important to identify familial mutations and associated implications: secondary malignancy prevention strategies for EOC patients, identification of other affected family members, and implementation of risk‐reduction strategies for affected family members. In this current cohort, this approach identified germline variants with a < 1% false‐negative rate, which flags a need for vigilance in minimizing false‐negative results, for instance by initiating a “gray zone” analysis particularly designed to flag small CNVs and to ensure gray zone cases undergo germline testing for CNV confirmation. Further investigations are required to best optimize and refine the tumor‐first genetic testing approach.

## Conflict of interest

Jeanna McCuaig is an employee of AstraZeneca since September 2023. She was not employed by them at any time during the development, initiation, or conduct of this study.

## Author contributions

ES: study planning, data collection, data interpretation, manuscript draft, and review. CM: data collection, data interpretation, manuscript draft and review. MC: data collection, data interpretation, manuscript draft and review. TS: study planning, data interpretation, manuscript draft and review. RK: study planning, data interpretation, manuscript review. JM: study planning, manuscript review. BC: study planning, manuscript review. LR: data collection, manuscript review. CS: data collection, manuscript review. SF: study planning, data interpretation, manuscript review. LH: data interpretation, manuscript review. TM: data interpretation, manuscript review. MB: study planning, supervision, data interpretation, manuscript review.

## Supporting information


**Data S1.** Overview of the Genetic Testing Performed.

**Fig. S1.** Overview of patient care trajectory from study enrolment to follow‐up period.

**Fig. S2.** Variant description (A) Somatic Tier III variants (*n* = 58) (B) Germline variants of uncertain significance (VUS) (*n* = 35).
