# Supplementary material for: Tumor and germline testing with next generation sequencing in epithelial ovarian cancer: a prospective paired comparison using an 18‐gene panel
Source: Mol Oncol. 2025 Oct 5;20(3):838–49. doi: 10.1002/1878-0261.70136 (PMC13042983; doi:10.1002/1878-0261.70136)
Supplement: Supplementary file 1 — Data S1. Overview of the Genetic Testing Performed. [file MOL2-20-838-s002.docx]

# SUPPLEMENTARY MATERIAL

**Supplementary Table 1. Overview of the Genetic Testing Performed**

| **Assay** | **Lab** | **Specimen** | **Panel description** | **Clinical Significance** |
| --- | --- | --- | --- | --- |
| NGS germline panel test | UHN Genome Diagnostics | Blood | NGS-based assay  18 genes: *BRCA1, BRCA2, MLH1, MSH2, MSH6, PMS2, EPCAM, RAD51C, RAD51D, BRIP1, ATM, BARD1, CDH1, CHEK2, PALB2, PTEN, STK11, TP53* | - Familial information, opportunity for risk-reduction in at-risk family members - Anticipate benefit of PARPi maintenance - Guide surveillance for secondary malignancies |
| NGS tumor panel test |  | Tumor |  | - Anticipate benefit of PARPi maintenance - Highlight subgroup of patients to forego germline testing |
| HRD tumor test | Myriad Genetics | Tumor | NGS-based assay to determine GIS* | - Anticipate benefit of PARPi maintenance |

*GIS: genomic instability score, NGS: next generation sequencing, PARPi: inhibitors of the enzyme poly ADP ribose polymerase, UHN: University Health Network; *GIS score is based on loss of heterozygosity, telomeric allelic imbalance and large-scale state transitions.*

**Supplementary Table 2. Reasons for not undergoing Myriad testing (n=33)**

| Unknown | 11 |
| --- | --- |
| Did not provide consent | 1 |
| Did not meet criteria | 3 |
| Lack of tissue | 1 |
| No surgery | 15 |
| Unsuccessful surgery | 2 |

# SUPPORTING INFORMATION

**Supplementary Table 1. Overview of the Genetic Testing Performed**

*GIS: genomic instability score, NGS: next generation sequencing, PARPi: inhibitors of the enzyme poly ADP ribose polymerase, UHN: University Health Network; GIS score is based on loss of heterozygosity, telomeric allelic imbalance and large-scale state transitions.*

**Supplementary Table 2. Reasons for not undergoing HRD Myriad testing (n=33)**

**Supplementary Figure 1.** **Overview of patient care trajectory from study enrolment to follow up period**

*eFHQ: electronic family history questionnaire, EOC: epithelial ovarian cancer, HRD: homologous recombinant deficiency, IR: interventional radiology, NACT: neoadjuvant chemotherapy, NGS: next generation sequencing, , PARPi: inhibitors of the enzyme poly ADP ribose polymerase, UHN university Health Network.*

**Supplementary Figure 2. Variant description A. Somatic Tier III variants (n=58) B. Germline variants of uncertain significance (VUS) (n=35)**

*n: number, VUS: variant of uncertain significance. Variants were classified according to scheme of Richards et al 2015[21]*
