# Supplementary figures and images for: Tumor and germline testing with next generation sequencing in epithelial ovarian cancer: a prospective paired comparison using an 18‐gene panel
Source: Mol Oncol. 2025 Oct 5;20(3):838–49. doi: 10.1002/1878-0261.70136 (PMC13042983; doi:10.1002/1878-0261.70136)

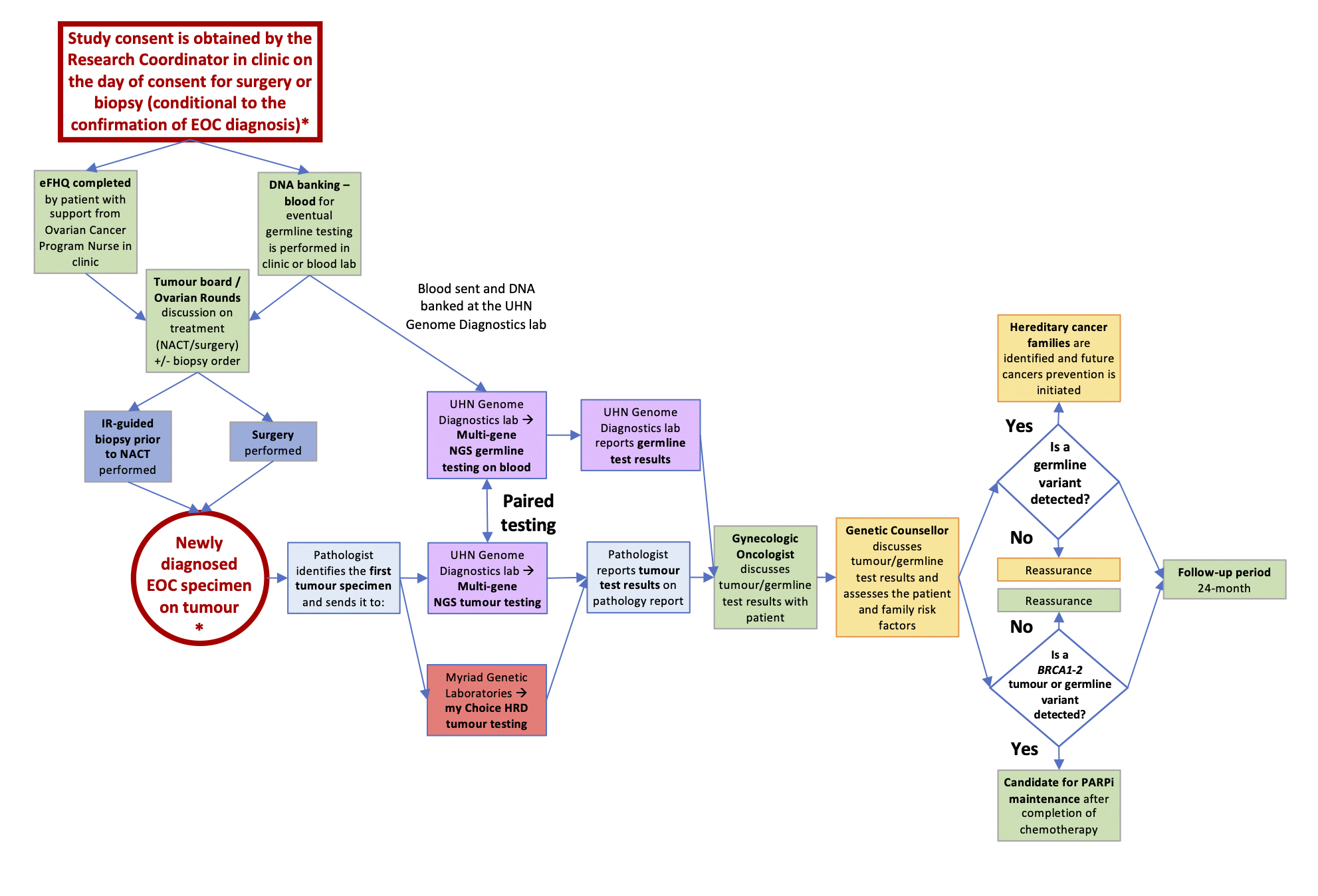

Supplement: Supplementary file 2 — Fig. S1. Overview of patient care trajectory from study enrolment to follow‐up period. [file MOL2-20-838-s001.jpg]

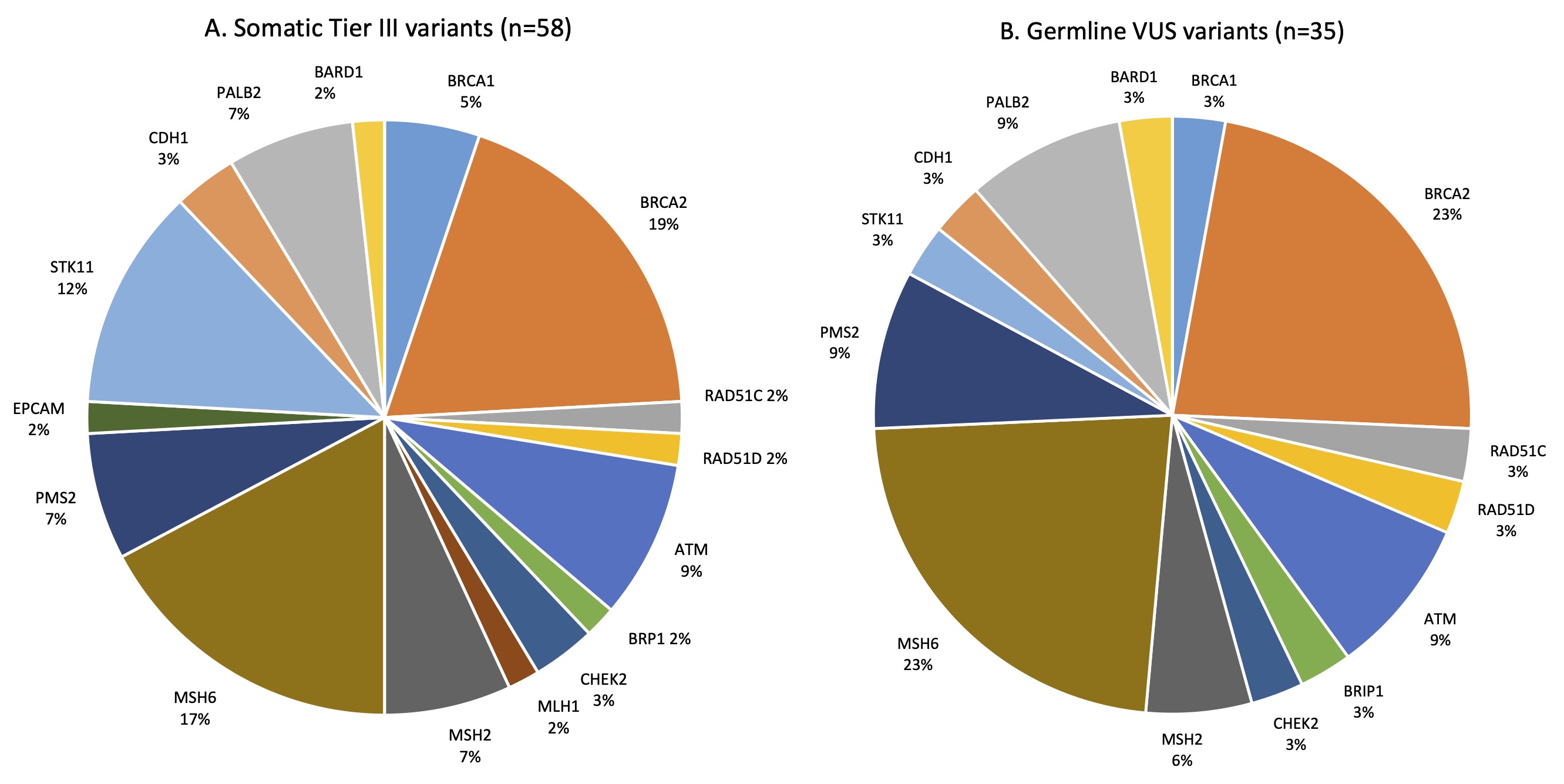

Supplement: Supplementary file 3 — Fig. S2. Variant description (A) Somatic Tier III variants (n = 58) (B) Germline variants of uncertain significance (VUS) (n = 35). [file MOL2-20-838-s003.jpg]
